# A note on improved *F*-expansion method combined with Riccati equation applied to nonlinear evolution equations

**DOI:** 10.1098/rsos.140038

**Published:** 2014-10-08

**Authors:** Md. Shafiqul Islam, Kamruzzaman Khan, M. Ali Akbar, Antonio Mastroberardino

**Affiliations:** 1 Department of Mathematics, Pabna University of Science and Technology, Pabna 6600, Bangladesh; 2 Department of Applied Mathematics, University of Rajshahi, Rajshahi 6205, Bangladesh; 3 School of Science, Penn State Erie, The Behrend College, Erie, PA 16563, USA

**Keywords:** improved -expansion method, modified Benjamin–Bona–Mahony equation, modified Korteweg-de Vries equation, exact solution, NLEEs

## Abstract

The purpose of this article is to present an analytical method, namely the improved *F*-expansion method combined with the Riccati equation, for finding exact solutions of nonlinear evolution equations. The present method is capable of calculating all branches of solutions simultaneously, even if multiple solutions are very close and thus difficult to distinguish with numerical techniques. To verify the computational efficiency, we consider the modified Benjamin–Bona–Mahony equation and the modified Korteweg-de Vries equation. Our results reveal that the method is a very effective and straightforward way of formulating the exact travelling wave solutions of nonlinear wave equations arising in mathematical physics and engineering.

## 2. Introduction

Nonlinear evolution equations (NLEEs) are widely used to describe many important phenomena and dynamical processes in mathematical physics and engineering. The investigation of exact solutions of NLEEs plays an important role in the study of these physical phenomena. In this paper, we highlight an analytical method, namely the improved *F*-expansion method, for finding exact solutions of NLEEs. Exact solutions provide a means to describe the salient features in various science, technology and engineering applications and can serve as a basis for perfecting and testing computer algebra software packages for solving NLEEs. It is significant that many equations of physics, chemistry and biology contain empirical parameters or empirical functions. Exact solutions allow researchers to design and run experiments by creating appropriate natural conditions in order to determine these parameters or functions.

However, not all equations of interest are solvable. Hence, it has become increasingly important to be familiar with all traditional and recently developed methods for solving these models and also to develop new methods. As a result, there has been a great amount of activity aimed at finding methods for solving not only NLEEs but also more general types of ordinary and partial differential equations. A list of several of the more well-known methods includes the solitary wave ansätz [1], the first integral method [2,3], the functional variable method [4,5], the Exp-function method [6–10], the modified simple equation method [11–13], the tanh–coth function method [14,15], the Kudryashov method [16,17], the exp(-*Φ*(*ξ*))-expansion method [18], the (*G*′/*G*)-expansion method [19–22], the homotopy perturbation method [23–27], the multiple exp-function method [28,29], Bernoulli sub-ODE method [30–32], the homotopy analysis method [33,34], the variational iteration method [35] and the *F*-expansion method [36,37].

In each of these aforementioned works, a variety of ansätz have been proposed for seeking travelling wave solutions of nonlinear differential equations. The choice of an appropriate ansätz is of great importance when using these analytical methods. Among those approaches, the proposed improved *F*-expansion method is a powerful tool to reveal more general solitons of NLEEs in mathematical physics and engineering. The main idea of this method is to express the travelling wave solutions of NLEEs in terms of functions that satisfy the Riccati equation *F*′(*ξ*)=*k*+*F*^2^(*ξ*).

The major advantage of the improved *F*-expansion method over the existing other methods is that it provides more new exact travelling wave solutions. To demonstrate the efficiency and reliability of this proposed method, the mBBM equation and the mKdV equation have been solved in which new solutions are found. It is important to note that analysis of convergence and stability for the numerical methods is required, a distinct disadvantage when compared with analytical methods that do not require such an analysis. Apart from the physical relevance, the closed-form solutions of NLEEs can assist the numerical solvers to measure up to the accuracy of their results and thus aid in the convergence analysis.

The rest of the article has been prepared as follows. In §2, the improved *F*-expansion method is discussed in detail. In §3, we apply this method to obtain solutions to the NLEES mentioned above. In §4, we provide graphical representations of some of the obtained solutions. Section 5 contains the conclusion of our study.

## 3. Algorithm of improved *F*-expansion method

In this section, we describe the main steps of the improved *F*-expansion method for finding exact travelling wave solutions of NLEEs. To begin, consider the well-known Riccati equation:

3.1 $$F^{\prime }(\xi )=k+F^{2}(\xi ),$$

where *F*=*F*(*ξ*) and the prime stands for derivatives with respect to *ξ*.

We now present the three cases of the general solutions of the Riccati equation (3.1).

*Case 1*. When *k*<0, the general solutions are

$$F_{1}=-\sqrt{-k}\tanh\left(\sqrt{-k}\;\xi \right)$$

and

$$F_{2}=-\sqrt{-k}\;\coth\left(\sqrt{-k}\xi \right).$$

*Case 2*. When *k*>0, the general solutions are

$$F_{4}=\sqrt{k}\;\tan\left(\sqrt{k}\;\xi \right)$$

and

$$F_{5}=-\sqrt{k}\;\cot\left(\sqrt{k}\;\xi \right).$$

*Case 3*. When *k*=0, the general solution is

$$F_{3}=-\frac{1}{\xi },$$

where *k* is the real parameter.

Now consider a general NLEE, say in two independent variables *x* and *t*,

3.2 $$P(u,u_{x},u_{y},u_{t},u_{xx},u_{xy},u_{xt},\ldots )=0,$$

where *u*=*u* (*x*, *y*, *t*) is an unknown function, *P* is a polynomial in *u* (*x*, *y*, *t*) and its partial derivatives in which the highest order partial derivatives and the nonlinear terms are involved and the subscripts stand for the partial derivatives. The foremost steps of the method are given below.

*Step 1.* We introduce the travelling wave transformation,

3.3 u(x,y,t)=u(ξ),ξ=x+y±λt,

where λ is the speed of the travelling wave and substitute this into equation (3.2), yielding the ordinary differential equation (ODE):

3.4 $$Q(u,u^{\prime },u^{\prime \prime },u^{\prime \prime \prime },\ldots )=0,$$

where *Q* is a polynomial of *u* and its derivatives and the superscripts specify the ordinary derivatives with respect to *ξ*.

*Step 2.* In many instances, equation (3.4) can be integrated term by term one or more times, yielding constants of integration, which can be set equal to zero for simplicity.

*Step 3.* We assume the travelling wave solution of equation (3.4) can be expressed by a polynomial in *F*(*ξ*) as follows:

3.5 $$u(\xi )=\sum_{i=0}^{N}\alpha_{i}{\left(m+F(\xi )\right)}^{i}+\sum_{i=1}^{N}\beta_{i}{\left(m+F(\xi )\right)}^{-i},$$

where *F*=*F*(*ξ*) satisfies the Riccati equation (3.1), *α*_*i*_(*i*=0,1,2,…,*N*), *β*_*i*_ (*i*=0,1,2,…,*N*), λ and *m* are constants to be determined later.

*Step 4.* The positive integer *N* can be determined by using homogeneous balance between the highest order derivatives and the nonlinear terms appearing in the ODE (3.4). If the degree of *u*(*ξ*) is *D*[*u*(*ξ*)]=*n*, then the degree of the other expressions will be given by

3.6 $$D\left[\frac{{\text{d}}^{p}u(\xi )}{\text{d}\xi^{p}}\right]=n+p\;\text{and}\;D\left[u^{p}{\left(\frac{{\text{d}}^{q}u(\xi )}{\text{d}\xi^{q}}\right)}^{s}\right]=np+s(n+p).$$

Therefore, we can find the value of *N* in equation (3.5), using equation (3.6).

*Step 5*. Substituting equation (3.5) into equation (3.4) together with the value of *N* obtained in step 4, we obtain polynomials in *F*(*ξ*). We set each coefficient of the resulting polynomial to zero, yielding an over-determined set of algebraic equations for *α*_*N*_, *β*_*N*_, *m* and λ.

*Step 6.* We suppose the values of the constants *α*_*N*_, *β*_*N*_, *m* and λ can be determined by solving the algebraic equations obtained in step 5. As the general solution of equation (3.1) is known to us, inserting the value of *α*_*N*_, *β*_*N*_, *m* and λ into equation (3.5) yields the general and new exact travelling wave solutions of the nonlinear partial differential equation (3.1).

## 4. Applications

**Example 4.1**

The modified Benjamin–Bona–Mahony (mBBM) equation.

The Benjamin–Bona–Mahony (BBM) equation $u_{t}+u_{x}+{au}^{n}u_{x}+{bu}_{xxt}=0$ is a well-known NLEE that models long waves in a nonlinear dispersive system. The solution of the BBM equation exhibits soliton-like behaviour. The BBM equation is used in the analysis of the surface waves of long wavelength in liquids, hydromagnetic waves in cold plasma, acoustic-gravity waves in compressible fluids and acoustic waves in harmonic crystals. When *n*=2, the BBM equation is called the modified BBM equation [3,38] and is given by

4.1 $$u_{t}+u_{x}+au^{2}u_{x}+bu_{xxt}=0,$$

where *a* and *b* are positive constants. This equation was introduced for modelling long waves of small amplitude in (1+1)-dimensions. We substitute the travelling wave transformation *u*(*x*, *t*)=*u*(*ξ*), *ξ*=*x*+λt into equation (4.1) and obtain the ODE

4.2 $$\lambda u^{\prime }+u^{\prime }+au^{2}u^{\prime }+b\lambda u^{\prime \prime \prime }=0.$$

Now integrating equation (4.2) with respect to *ξ* once and setting the constant of integration to zero, we obtain

4.3 $$b\lambda u^{\prime \prime }+\frac{a}{3}u^{3}+(\lambda +1)u=0.$$

Taking the homogeneous balance between the highest order nonlinear term *u*^3^ and the derivative term *u*′′ from equation (4.3), yields 3*N*=*N*+2, i.e. *N*=1.

Hence for *N*=1 equation (3.5) reduces to

4.4 $$u(\xi )=\alpha_{0}+\alpha_{1}(m+F(\xi ))+\beta_{1}{\left(m+F(\xi )\right)}^{-1}.$$

Now substituting equation (4.4) into equation (4.3), we obtain a polynomial in *F*(*ξ*). Setting the coefficients of the powers of *F*(*ξ*) to zero, we obtain the following system of algebraic equations:

$$\begin{array}{rl} & 6b\lambda \alpha_{1}+\alpha \alpha_{1}^{3}=0,\\ & 3\alpha \alpha_{0}\alpha_{1}^{2}+6\alpha \alpha_{1}^{3}+18b\lambda \alpha_{1}m=0,\\ & 6b\lambda \alpha_{1}k+3\alpha \alpha_{0}^{2}\alpha_{1}+15\alpha \alpha_{0}\alpha_{1}^{2}m+18b\lambda \alpha_{1}m^{2}+15\alpha \alpha_{1}^{3}m^{2}+3\lambda \alpha_{1}+3\alpha \alpha_{1}^{2}\beta_{1}+3\alpha_{1}=0,\\ & 20\alpha \alpha_{1}^{3}m^{3}+30\alpha \alpha_{0}\alpha_{1}^{2}m^{2}+12\alpha_{1}m+12\alpha \alpha_{1}^{2}m\beta_{1}+3\alpha_{0}+18b\lambda \alpha_{1}km+\alpha \alpha_{0}^{3}-6b\lambda \beta_{1}m+3\lambda \alpha_{0}\\ & \;+12\lambda \alpha_{1}m+6b\lambda \alpha_{1}m^{3}+6\alpha \alpha_{0}\alpha_{1}\beta_{1}+12\alpha \alpha_{0}^{2}\alpha_{1}m=0,\\ & 3\lambda \beta_{1}+18\alpha \alpha_{1}^{2}m^{2}\beta_{1}+3\alpha \alpha_{1}\beta_{1}^{2}+6b\lambda \beta_{1}k+18\alpha \alpha_{0}\alpha_{1}m\beta_{1}+18\alpha_{1}m^{2}+18b\lambda \alpha_{1}km^{2}+18\lambda \alpha_{1}m^{2}\\ & \;+3\alpha \alpha_{0}^{2}\beta_{1}+3\beta_{1}+9\alpha_{0}m+9\lambda \alpha_{0}m+15\alpha \alpha_{1}^{3}m^{4}+18\alpha \alpha_{0}^{2}\alpha_{1}m^{2}+3\alpha \alpha_{0}^{3}m+30\alpha \alpha_{0}\alpha_{1}^{2}m^{3}=0,\\ & -6b\lambda \beta_{1}km+12\alpha \alpha_{0}^{2}\alpha_{1}m^{3}+12\alpha_{1}m^{3}+18\alpha \alpha_{0}\alpha_{1}\beta_{1}m^{2}+6\beta_{1}m+6\alpha \alpha_{1}m\beta_{1}^{2}+9\lambda \alpha_{0}m^{2}+3\alpha \alpha_{0}\beta_{1}^{2}\\ & \;+6b\lambda \alpha_{1}km^{3}+15\alpha \alpha_{0}\alpha_{1}^{2}m^{4}+12\alpha \alpha_{1}^{2}m^{3}\beta_{1}+6\alpha \alpha_{1}^{3}m^{5}+9\alpha_{0}m^{2}+6\alpha \alpha_{0}^{2}\beta_{1}m+6\lambda \beta_{1}m+12\lambda \alpha_{1}m^{3}\\ & \;+3\alpha \alpha_{0}^{3}m^{2}=0\\ \text{and}\; & \alpha \beta_{1}^{3}+3\lambda \alpha_{1}m^{4}+\alpha \alpha_{1}^{3}m^{6}+6b\lambda \beta_{1}k^{2}+3\alpha_{0}m^{3}+3\lambda \beta_{1}m^{2}+3\alpha_{1}m^{4}+3\alpha \alpha_{0}^{2}\beta_{1}m^{2}+3\alpha \alpha_{1}^{2}m^{4}\beta_{1}\\ & \;+3\alpha \alpha_{1}m^{2}\beta_{1}^{2}+\alpha \alpha_{0}^{3}m^{3}+3\lambda \alpha_{0}m^{3}+3\alpha \alpha_{0}\beta_{1}^{2}m+3\alpha \alpha_{0}\alpha_{1}^{2}m^{5}+3\beta_{1}m^{2}+3\alpha \alpha_{0}^{2}\alpha_{1}m^{4}\\ & \;+6\alpha \alpha_{0}\alpha_{1}m^{3}\beta_{1}=0. \end{array}$$

Solving the above system of equations for *α*_0_, *α*_1_, *β*_1_, *m* and λ, we obtain the following values:

*Set-1*:

$$m=0,\lambda =-\frac{1}{1+2bk},\alpha_{0}=0,\alpha_{1}=0,\beta_{1}=\pm \frac{\sqrt{6b}\;k}{\sqrt{a(1+2bk)}}.$$

*Set-2*:

$$m=m,\lambda =-\frac{1}{1+2bk},\alpha_{0}=\mp \frac{\sqrt{6b}m}{\sqrt{a(1+2bk)}},\alpha_{1}=\pm \frac{\sqrt{6b}}{\sqrt{a(1+2bk)}},\beta_{1}=0.$$

*Set-3*:

$$m=0,\lambda =\frac{1}{8bk+1},\alpha_{0}=0,\alpha_{1}=\pm \frac{\sqrt{6b}}{\sqrt{a(8bk+1)}},\beta_{1}=\mp \frac{\sqrt{6b}k}{\sqrt{ab(8bk+1)}}.$$

*Set-4*:

$$m=0,\lambda =\frac{1}{4bk+1},\alpha_{0}=0,\alpha_{1}=\pm \frac{\sqrt{-(2bk-6)ab}}{(4bk-1)a},\beta_{1}=\mp \frac{6bk}{\sqrt{-(24bk-6)ab}}.$$

*Set-5*:

$$\begin{array}{rl} m & =\pm \frac{1}{6}\frac{\sqrt{-6b(1+2bk)}}{b},\lambda =-\frac{1}{1+2bk},\alpha_{0}=\mp \frac{1}{\sqrt{-a}},\alpha_{1}=0\\ \beta_{1} & =\pm \frac{4bk-1}{\sqrt{-a}\sqrt{-6b(1+2bk)}}. \end{array}$$

*Set-6*:

$$\begin{array}{rl} m & =\pm \frac{\sqrt{ab(1+2bk)}\alpha_{0}}{\sqrt{6}b},\lambda =-\frac{1}{1+2bk},\alpha_{0}=\alpha_{0},\alpha_{1}=0\\ \beta_{1} & =\mp \frac{(2bka\alpha_{0}^{2}+a\alpha_{0}^{2}+6bk)\;}{\sqrt{6ab(1+2bk)}}. \end{array}$$

*Case 1.* When *k*<0, we get the following hyperbolic trigonometric solutions.

*Family-1*:

$$u_{1,2}(\xi )=\pm \frac{\sqrt{6b}\sqrt{-k}}{\sqrt{a(1+2bk)}}\coth\left(\sqrt{-k}\xi \right)$$

and

$$u_{3,4}(\xi )=\pm \frac{\sqrt{6b}\sqrt{-k}}{\sqrt{a(1+2bk)}}\tanh\left(\sqrt{-k}\xi \right),$$

where *ξ*=*x*−*t*/(1+2*bk*).

*Family-2*:

$$u_{5,6}(\xi )=\mp \frac{\sqrt{6b}\sqrt{-k}}{\sqrt{a(1+2bk)}}\tanh\left(\sqrt{-k}\xi \right)$$

and

$$u_{7,8}(\xi )=\mp \frac{\sqrt{6b}\sqrt{-k}}{\sqrt{a(1+2bk)}}\coth\left(\sqrt{-k}\xi \right),$$

where *ξ*=*x*−*t*/(1+2*bk*).

*Family-3*:

$$u_{9,10}(\xi )=\mp \frac{b\sqrt{6}\sqrt{-k}\left(1+\tanh^{2}\left(\sqrt{-k}\xi \right)\right)}{\sqrt{a(8kb+1)}}\coth\left(\sqrt{-k}\xi \right),$$

and

$$u_{11,12}(\xi )=\mp \frac{b\sqrt{6}\sqrt{-k}\left(1+\coth^{2}\left(\sqrt{-k}\xi \right)\right)}{\sqrt{a(8kb+1)}}\tanh\left(\sqrt{-k}\xi \right),$$

where *ξ*=*x*−*t*/(8*bk*+1).

*Family-4*:

$$u_{13,14}(\xi )=\mp \frac{2\sqrt{-6bk}}{\sqrt{-a(4bk-1)}}\csc h\;\left(2\sqrt{-k}\xi \right)$$

and

$$u_{15,16}(\xi )=\pm \frac{2I\sqrt{-6bk}}{\sqrt{-a(4bk-1)}}\sec h\;\left(2\sqrt{-k}\xi \right),$$

where *ξ*=*x*+*t*/(4*bk*−1).

*Family-5*:

$$u_{17,18}(\xi )=\pm \frac{b\left(6bk\pm \sqrt{6}\sqrt{-b\left(1+2bk\right)}\sqrt{-k}\tanh\left(\sqrt{-k}\xi \right)\right)\sqrt{6}}{\sqrt{-a}\sqrt{-b(1+2bk)}\left(\sqrt{6}\sqrt{-b(1+2bk)}\mp 6b\sqrt{-k}\tanh\left(\sqrt{-k}\xi \right)\right)}$$

and

$$u_{19,20}(\xi )=\pm \frac{b\left(6bk\pm \sqrt{6}\sqrt{-b\;\left(1+2bk\right)}\sqrt{-k}\coth\left(\sqrt{-k}\xi \right)\right)\sqrt{6}}{\sqrt{-a}\sqrt{-b(1+2bk)}\left(\sqrt{6}\sqrt{-b(1+2bk)}\mp 6b\sqrt{-k}\coth\left(\sqrt{-k}\xi \right)\right)},$$

where *ξ*=*x*−*t*/(1+2*bk*).

*Family-6*:

$$u_{21,22}(\xi )=-\left(\frac{6b\left(\sqrt{6}bk\pm \alpha_{0}\sqrt{ab(1+2bk)}\sqrt{-k}\tanh\left(\sqrt{-k}\xi \right)\right)}{\sqrt{ab(1+2bk)}\left(\alpha_{0}\sqrt{6}\sqrt{ab\left(1+2bk\right)}\mp 6b\sqrt{-k}\tanh\left(\sqrt{-k}\xi \right)\right)}\right)$$

and

$$u_{23,24}(\xi )=-\left(\frac{6b\left(\sqrt{6}bk\pm \alpha_{0}\sqrt{ab\left(1+2bk\right)}\sqrt{-k}\coth\left(\sqrt{-k}\xi \right)\right)}{\sqrt{ab(1+2bk)}\left(\alpha_{0}\sqrt{6}\sqrt{ab(1+2bk)}\mp 6b\sqrt{-k}\coth\left(\sqrt{-k}\xi \right)\right)}\right),$$

where *ξ*=*x*−*t*/(1+2*bk*).

*Case 2.* When *k*>0, we get the following trigonometric solutions.

*Family-7*:

$$u_{25,26}(\xi )=\pm \frac{\sqrt{6bk}}{\sqrt{a(1+2bk)}}\cot\left(\sqrt{k}\xi \right)$$

and

$$u_{27,28}(\xi )=\mp \frac{\sqrt{6bk}}{\sqrt{a(1+2bk)}}\tan\left(\sqrt{k}\xi \right),$$

where *ξ*=*x*−*t*/(1+2*bk*)

*Family-8*:

$$u_{29,30}(\xi )=\pm \frac{\sqrt{6bk}}{\sqrt{a(1+2bk)}}\tan\left(\sqrt{k\xi }\right)$$

and

$$u_{31,32}(\xi )=\mp \frac{\sqrt{6bk}\;}{\sqrt{a(1+2bk)}}\cot\left(\sqrt{k\xi }\right),$$

where *ξ*=*x*−*t*/(1+2*bk*).

*Family-9*:

$$u_{33,34}(\xi )=\pm \frac{b\sqrt{6k}(\tan^{2}(\sqrt{k}\xi )-1)}{\sqrt{ab(8bk+1)}}\cot\left(\sqrt{k}\xi \right)$$

and

$$u_{35,36}(\xi )=\mp \frac{b\sqrt{6k}(\cot^{2}(\sqrt{k}\xi )-1)}{\sqrt{ab(8bk+1)}}\tan\left(\sqrt{k}\xi \right),$$

where *ξ*=*x*−*t*/(8*bk*+1).

*Family-10*:

$$u_{37,38}(\xi )=\mp \frac{2I\sqrt{6bk}}{\sqrt{a(4bk-1)}}\csc\left(2\sqrt{k}\xi \right)$$

and

$$u_{39,40}(\xi )=\pm \frac{2I\sqrt{6bk}}{\sqrt{a(4bk-1)}}\sec\left(2\sqrt{k}\xi \right),$$

where *ξ*=*x*+*t*/(4*bk*−1).

*Family-11*:

$$u_{41,42}(\xi )=\pm \left(\frac{b\left(6bk\mp \sqrt{6}\sqrt{-b(1+2bk)}\sqrt{k}\tan\left(\sqrt{k}\xi \right)\right)\sqrt{6}}{\sqrt{-a}\sqrt{-b(1+2bk)}\left(\sqrt{6}\sqrt{-b(1+2bk)}\pm 6b\sqrt{k}\tan\left(\sqrt{k}\xi \right)\right)}\right)$$

and

$$u_{43,44}(\xi )=\pm \left(\frac{b\left(6bk\pm \sqrt{6}\sqrt{-b(1+2bk)}\sqrt{k}\cot\left(\sqrt{k}\xi \right)\right)\sqrt{6}}{\sqrt{-a}\sqrt{-b(1+2bk)}\left(\sqrt{6}\sqrt{-b\;(1+2bk)}\mp 6b\sqrt{k}\cot\left(\sqrt{k}\xi \right)\right)}\right),$$

where *ξ*=*x*−*t*/(1+2*bk*).

*Family-12*:

$$u_{45,46}(\xi )=\pm \left(\frac{6b\left(\sqrt{6}bk\mp \alpha_{0}\sqrt{ab(1+2bk)}\sqrt{k}\tan\left(\sqrt{k}\xi \right)\right)}{\sqrt{ab(1+2bk)}\left(\alpha_{0}\sqrt{6}\sqrt{ab(1+2bk)}\pm 6b\sqrt{k}\tan\left(\sqrt{k}\xi \right)\right)}\right)$$

and

$$u_{47,48}(\xi )=-\left(\frac{6b\left(\sqrt{6}bk\pm \alpha_{0}\sqrt{ab(1+2bk)}\sqrt{k}\cot\left(\sqrt{k}\xi \right)\right)}{\sqrt{ab(1+2bk)}\left(\alpha_{0}\sqrt{6}\sqrt{ab(1+2bk)}\mp 6b\sqrt{k}\cot\left(\sqrt{k}\xi \right)\right)}\right),$$

where *ξ*=*x*−*t*/(1+2*bk*).

*Case 3.* When *k*=0, we get the following solutions.

*Family-13*:

$$u_{49,50}(\xi )=\mp \left(\frac{\sqrt{6b}}{\sqrt{a}\xi }\right),$$

where *ξ*=*x*−*t*.

*Family-14*:

$$u_{51,52}(\xi )=\mp \frac{\sqrt{6b}}{\sqrt{a}\xi },$$

where *ξ*=*x*+*t*.

*Family-15*:

$$u_{53,54}(\xi )=\pm \frac{\sqrt{6b}}{\sqrt{a\;}\xi },$$

where *ξ*=*x*−*t*.

*Family-16*:

$$u_{55,56}(\xi )=\frac{6b}{\sqrt{-a}\left(\sqrt{6}\sqrt{-b}\xi \mp 6b\right)},$$

where *ξ*=*x*−*t*.

*Family-17*:

$$u_{57,58}(\xi )=\mp \left(\frac{6\alpha_{0}b}{\sqrt{6}\sqrt{ab}\alpha_{0}\xi \mp 6b}\right),$$

where *ξ*=*x*−*t*.

**Remark**

All of these solutions have been verified with Maple by substituting them into the original equations.

**Example 4.2**

The modified Korteweg–de Vries (mKdV) equation.

In this section, we consider the mKdV equation given by

4.5 $$u_{t}-u^{2}u_{x}+\delta \;u_{xxx}=0,$$

where *δ* is a non-zero constant. The mKdV equation is similar to the KdV equation in that both are completely integrable and each has infinitely many conserved quantities. The mKdV equation appears in the study of electric circuits and multi-component plasmas [39,40].

We substitute the travelling wave transformation *u*(*x*,*t*)=*u*(*ξ*), *ξ*=*x*+λt into equation (4.5) and obtain the ODE:

4.6 $$\lambda u^{\prime }-u^{2}u^{\prime }+\delta u^{\prime \prime \prime }=0.$$

Now integrating equation (4.6) with respect to *ξ* once and setting the constant of integration to zero, we obtain

4.7 $$\delta u^{\prime \prime }-\frac{u^{3}}{3}+\lambda u=0.$$

Taking the homogeneous balance between the highest order nonlinear term *u*^3^ and the derivative term *u*′′ from equation (4.3), yields 3*N*=*N*+2, i.e. *N*=1.

Hence for *N*=1 equation (2.5) reduces to

4.8 $$u(\xi )=\alpha_{0}+\alpha_{1}(m+F(\xi ))+\beta_{1}{\left(m+F(\xi )\right)}^{-1}.$$

Now substituting equation (4.8) into equation (4.7), we get a polynomial in *F*(*ξ*). Setting the coefficients of the powers of *F*(*ξ*) to zero, we obtain the following system of algebraic equations:

$$\begin{array}{rl} & 6\delta \alpha_{1}-\alpha_{1}^{3}=0,\\ & 18\delta \alpha_{1}m-3\alpha_{0}\alpha_{1}^{2}-6\alpha_{1}^{3}m=0,\\ & 6\delta \alpha_{1}k-15\alpha_{1}^{3}m^{2}-3\alpha_{1}^{2}\beta_{+}18\delta \alpha_{1}m^{2}+3\lambda \alpha_{1}-3\alpha_{0}^{2}\alpha_{1}-15\alpha_{0}\alpha_{1}^{2}m=0,\\ & 12\lambda \alpha_{1}m-12\alpha_{1}^{2}m\beta_{1}-6\delta \beta_{1}m-30\alpha_{0}\alpha_{1}^{2}m^{2}+18\delta \alpha_{1}km+3\lambda \alpha_{0}-20\alpha_{1}^{3}m^{3}-6\alpha_{0}\alpha_{1}\beta_{1}+6\delta \alpha_{1}m^{3}\\ & \;-12\alpha_{0}^{2}\alpha_{1}m-\alpha_{0}^{3}=0,\\ & -15\alpha_{1}^{3}m^{4}-18\alpha_{1}^{2}m^{2}\beta_{1}+9\lambda \alpha_{0}m-18\alpha_{0}\alpha_{1}m\beta_{1}+18\lambda \alpha_{1}m^{2}+18\delta \alpha_{1}km^{2}-3\alpha_{1}\beta_{1}^{2}+6\delta \beta_{1}k\\ & \;-18\alpha_{0}^{2}\alpha_{1}m^{2}-3\alpha_{0}^{3}m+3\lambda \beta_{1}-30\alpha_{0}\alpha_{1}^{2}m^{3}-3\alpha_{0}^{2}\beta_{1}=0,\\ & -6\alpha_{0}^{2}\beta_{1}m-18\alpha_{0}\alpha_{1}\beta_{1}m^{2}-12\alpha_{1}^{2}m^{3}\beta_{1}+6\lambda \beta_{1}m-15\alpha_{0}\alpha_{1}^{2}m^{4}+12\lambda \alpha_{1}m^{3}+9\lambda \alpha_{0}m^{2}-6\alpha_{1}m\beta_{1}^{2}\\ & \;-3\alpha_{0}^{3}m^{2}+6\delta \alpha_{1}km^{3}-6\delta \beta_{1}km-3\alpha_{0}\beta_{1}^{2}-12\alpha_{0}^{2}\alpha_{1}m^{3}-6\alpha_{1}^{3}m^{5}=0\\ \text{and}\; & 6\delta \beta_{1}k^{2}-3\alpha_{1}^{2}m^{4}\beta_{1}-3\alpha_{1}m^{2}\beta_{1}^{2}-\alpha_{0}^{3}m^{3}-6\alpha_{0}\alpha_{1}m^{3}\beta_{1}+3\lambda \alpha_{0}m^{3}-3\alpha_{0}^{2}\beta_{1}m^{2}+3\lambda \alpha_{1}m^{4}+3\lambda \beta_{1}m^{2}\\ & \;-\alpha_{1}^{3}m^{6}-3\alpha_{0}\beta_{1}^{2}m-3\alpha_{0}\alpha_{1}^{2}m^{5}-\beta_{1}^{3}-3\alpha_{0}^{2}\alpha_{1}m^{4}=0. \end{array}$$

Solving the above system of equations for *α*_0_, *α*_1_, *β*_1_, *m* and λ, we get the following values:

*Set-1*:

$$m=0,\lambda =-2\delta k,\alpha_{0}=0,\alpha_{1}=0,\beta_{1}=\pm \sqrt{6\delta }k.$$

*Set-2*:

$$m=\pm \frac{1}{\sqrt{6\delta }}\cdot \alpha_{0},\lambda =-2\delta k,\alpha_{0}=\alpha_{0},\alpha_{1}=0,\beta_{1}=\mp \frac{\left(\alpha_{0}^{2}+6\delta k\right)}{\sqrt{6\delta }}.$$

*Set-3*:

$$m=m,\lambda =-2\delta k,\alpha_{0}=\mp \sqrt{6\delta }m,\alpha_{1}=\pm \sqrt{6\delta },\beta_{1}=0.$$

*Set-4*:

$$m=0,\lambda =-2\delta k\pm 6\delta k,\alpha_{0}=0,\alpha_{1}=\pm \sqrt{6\delta },\beta_{1}=\pm \sqrt{6\delta }k.$$

*Case 1.* When *k*<0, we get the following hyperbolic trigonometric solutions.

*Family-1*:

$$u_{1,2}(\xi )=\pm \sqrt{6\delta }\sqrt{-k}\coth\left(\sqrt{-k}\xi \right)$$

and

$$u_{3,4}(\xi )=\pm \sqrt{6\delta }\sqrt{-k}\tanh\left(\sqrt{-k}\xi \right),$$

where *ξ*=*x*−2*δ*kt.

*Family-2*:

$$u_{5,6}(\xi )=\mp \left(\frac{6(\alpha_{0}\sqrt{-k}\tanh\left(\sqrt{-k}\xi \right)\sqrt{\delta }\pm \sqrt{6}\delta k)}{\sqrt{6}\alpha_{0}\mp 6\sqrt{-k}\tanh\left(\sqrt{-k}\xi \right)\sqrt{\delta }}\right)$$

and

$$u_{7,8}(\xi )=\mp \left(\frac{6\left(\alpha_{0}\sqrt{-k}\coth\left(\sqrt{-k}\xi \right)\sqrt{\delta }\pm \sqrt{6}\delta k\right)}{\sqrt{6}\alpha_{0}\mp 6\sqrt{-k}\coth\left(\sqrt{-k}\xi \right)\sqrt{\delta }}\right),$$

where *ξ*=*x*−2*δ*kt.

*Family-3*:

$$u_{9,10}(\xi )=\mp \sqrt{6\delta }\sqrt{-k}\tanh\left(\sqrt{-k}\xi \right)$$

and

$$u_{11,12}(\xi )=\mp \sqrt{6\delta }\sqrt{-k}\coth\left(\sqrt{-k}\xi \right),$$

where *ξ*=*x*−2*δ*kt.

*Family-4*:

$$u_{13,14}(\xi )=\pm \sqrt{6\delta }\sqrt{-k}\csc h\left(\sqrt{-k}\xi \right)\sec h\left(\sqrt{-k}\xi \right),$$

where *ξ*=*x*+(−2*δk*±6*δ*)*t*.

*Case 2.* When *k*>0, we get the following trigonometric solutions.

*Family-5*:

$$u_{15,16}(\xi )=\pm \sqrt{6\delta k}\cot\left(\sqrt{k}\xi \right)$$

and

$$u_{17,18}(\xi )=\mp \sqrt{6\delta k}\tan\left(\sqrt{k}\xi \right),$$

where *ξ*=*x*−2*δ* kt.

*Family-6*:

$$u_{19,20}(\xi )=\pm \left(\frac{6\left(\alpha_{0}\sqrt{k}\tan\left(\sqrt{k}\xi \right)\sqrt{\delta }\mp \sqrt{6}\delta k\right)}{\sqrt{6}\alpha_{0}\pm 6\sqrt{k}\tan\left(\sqrt{k}\xi \right)\sqrt{\delta }}\right)$$

and

$$u_{21,22}(\xi )=\mp \left(\frac{6\left(\alpha_{0}\sqrt{k}\cot\left(\sqrt{k}\xi \right)\sqrt{\delta }\pm \sqrt{6}\delta \;k\right)}{\sqrt{6}\alpha_{0}\mp 6\sqrt{k}\cot\left(\sqrt{k}\xi \right)\sqrt{\delta }}\right),$$

where *ξ*=*x*−2*δ*kt.

*Family-7*:

$$u_{23,24}(\xi )=\pm \sqrt{6\delta k}\tan\left(\sqrt{k}\xi \right)$$

and

$$u_{25,26}(\xi )=\mp \sqrt{6\delta k}\cot\left(\sqrt{k}\xi \right),$$

where *ξ*=*x*−2*δ*kt.

*Family-8*:

$$u_{27,28}(\xi )=\pm \sqrt{6\delta k}\csc\left(\sqrt{k}\xi \right)\sec\left(\sqrt{k}\xi \right),$$

where *ξ*=*x*+(−2*δ*k±6*δ*)*t*.

*Case 3.* When *k*=0, we get the following solutions.

*Family-9*:

$$u_{29,30}(\xi )=\mp \left(\frac{6\alpha_{0}\sqrt{\delta }}{\sqrt{6}\alpha_{0}\xi \mp 6\sqrt{\delta }}\right),$$

where *ξ*=*x*.

*Family-10*:

$$u_{31,32}(\xi )=\mp \frac{\sqrt{6\delta }}{\xi },$$

where *ξ*=*x*.

*Family-11*:

$$u_{33,34}(\xi )=\mp \frac{\sqrt{6\delta }}{\xi },$$

where *ξ*=*x*.

**Remark**

All of these solutions have been verified with Maple by substituting them into the original equations.

## 5. Graphical representation of the obtained solutions

**Figure 1.**
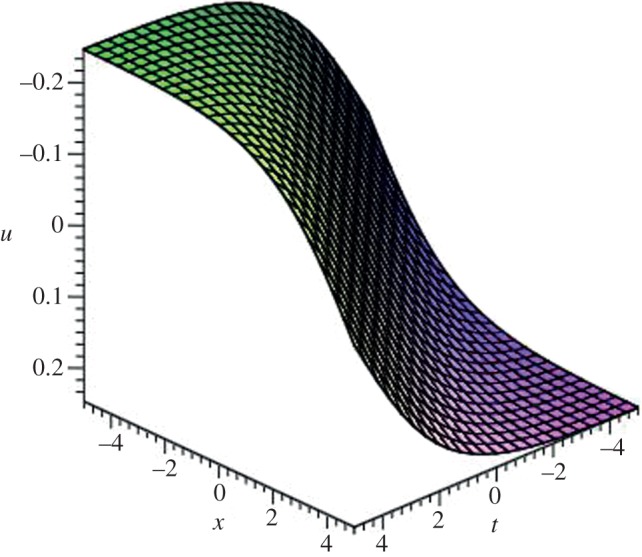
Kink-shaped soliton solution *u*_3_(*ξ*) of mBBM equation for *a*=1, *b*=0.10 and *k*=−0.10.

**Figure 2.**
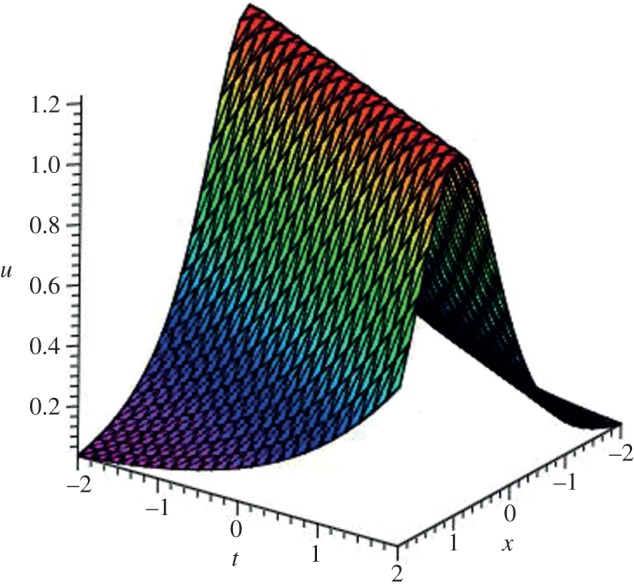
Bell-shaped soliton solution *u*_13_(*ξ*) of mBBM equation for *a*=2, *b*=0.50 and *k*=−0.50.

**Figure 3.**
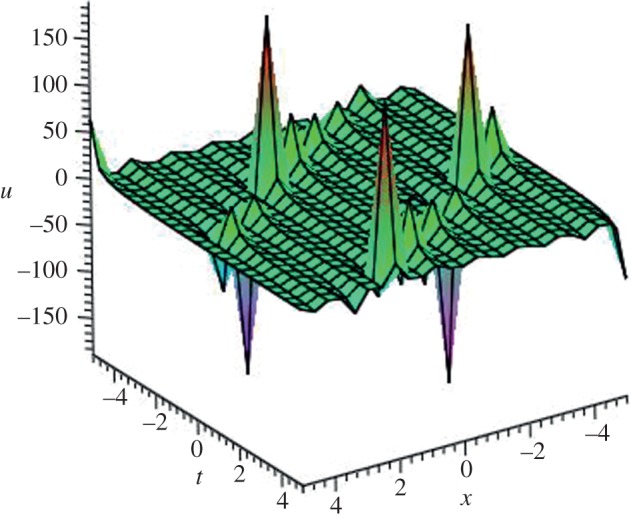
Periodic solution *u*_29_(*ξ*) of mBBM equation for *a*=1, *b*=1 and *k*=7.

**Figure 4.**
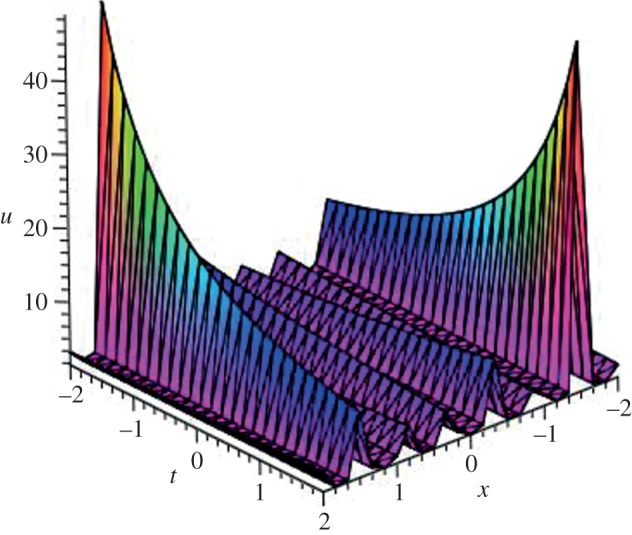
Periodic solution *u*_39_(*ξ*) of mBBM equation for *a*=3, *b*=7 and *k*=7.

**Figure 5.**
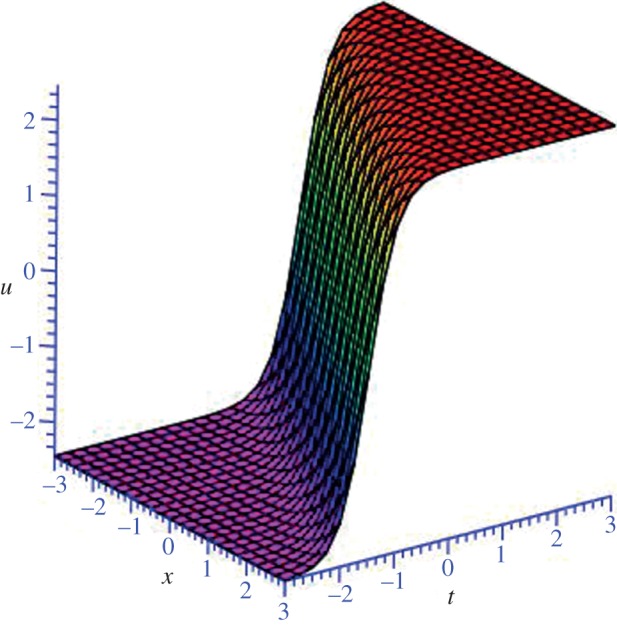
Kink-shaped soliton solution *u*_3_(*ξ*) of mKdV equation for *δ*=1 and *k*=−1.

**Figure 6.**
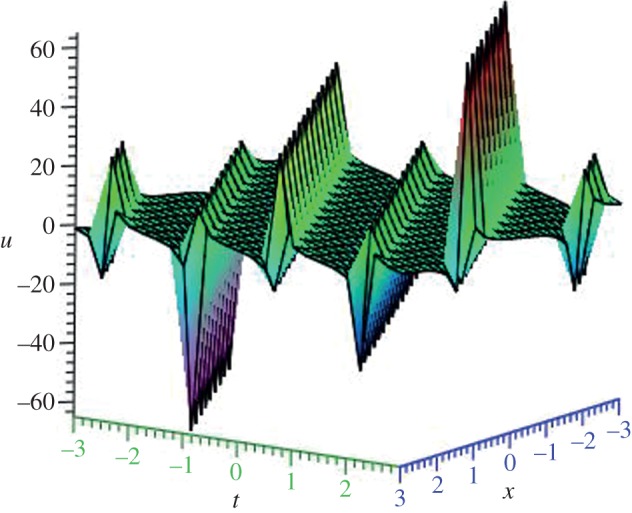
Periodic solution *u*_18_(*ξ*) of mKdV equation for *δ*=1 and *k*=1.

Using mathematical software Maple, three-dimensional plots of some obtained solutions are shown in figures 1–6 to visualize the underlying features of the exact travelling wave solutions. In particular, three-dimensional profiles for the solutions of mBBM equation are represented in figures 1–4 and that of the mKdV equation are shown in figures 5 and 6.

## 6. Conclusion

In this paper, we have used the improved *F*-expansion method to seek exact solutions of mBBM and mKdV equations and have found new solutions. Each of the obtained solutions, given in terms of hyperbolic solutions, trigonometric solutions and rational solutions, contains an explicit function of the variables in the considered equation. The performance of the improved *F*-expansion method confirms that it is a reliable and effective technique for finding exact solutions for a large class of problems in mathematical physics and engineering and can also be extended to other types of NLEEs. A similar study for solving other models, like Burgers equation, Fisher’s equation, Schrödinger equation, Sine-Gordon equation, Klein Gordon equation, etc., that arise in mathematical physics and engineering is a possible future direction.
